# Supplementary material for: Shared Treg dysregulation underlies psoriasis vulgaris and atopic dermatitis through the PHF19-PRC2 epigenetic axis
Source: Front Immunol. 2026 Aug 20;17:1932788. doi: 10.3389/fimmu.2026.1932788 (PMC13538097; doi:10.3389/fimmu.2026.1932788)
Supplement: Supplementary file 3 [file DataSheet3.zip › Additional files/Additional file 8/65.HLA-B.leaveoneout.pdf]

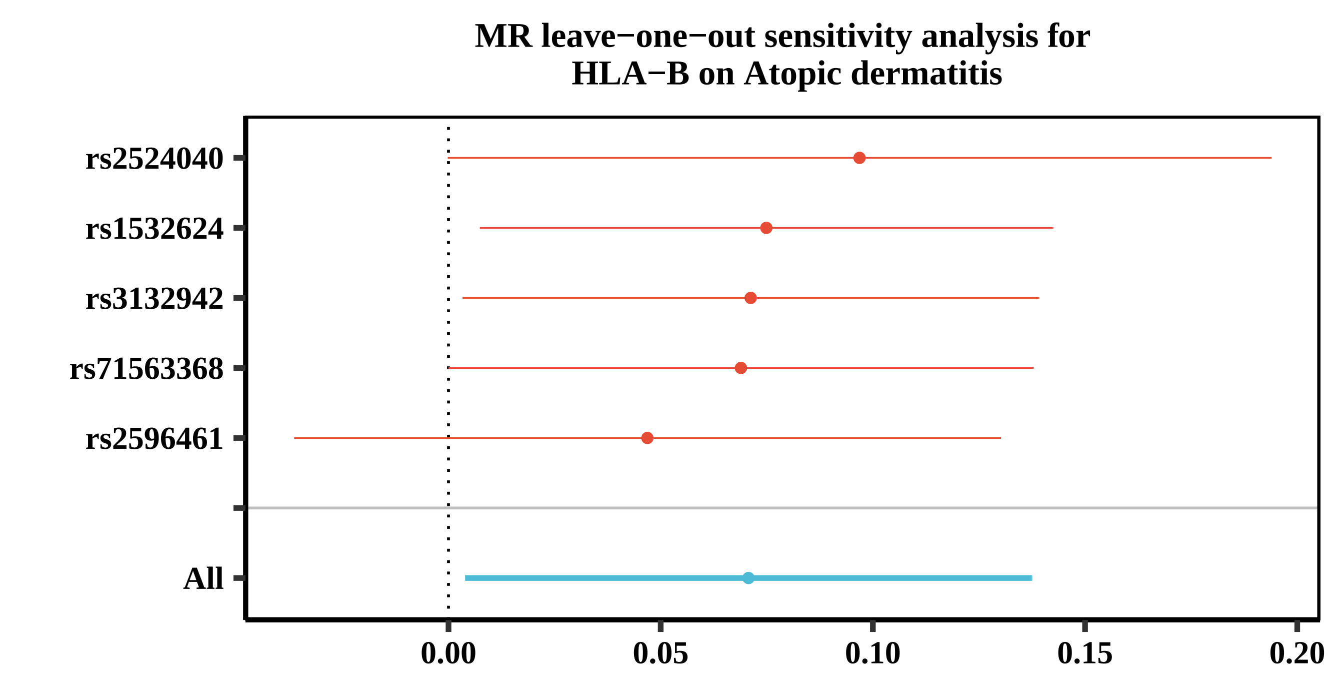
MR leave−one−out sensitivity analysis for
HLA−B on Atopic dermatitis
rs2524040
rs1532624
rs3132942
rs71563368
rs2596461
All
0.00
0.05
0.10
0.15
0.20
